# The Effect of Different Porogens on Porous PMMA Microspheres by Seed Swelling Polymerization and Its Application in High-Performance Liquid Chromatography

**DOI:** 10.3390/ma11050705

**Published:** 2018-04-29

**Authors:** Bing Yu, Tingting Xue, Long Pang, Xiulan Zhang, Youqing Shen, Hailin Cong

**Affiliations:** 1 Institute of Biomedical Materials and Engineering, College of Chemistry and Chemical Engineering, College of Materials Science and Engineering, Qingdao University, Qingdao 266071, China; yubingqdu@yahoo.com (B.Y.); xuetingt@126.com (T.X.); panglongstuta@163.com (L.P.); 18765916806@139.com (X.Z.); amnano@163.com (Y.S.); 2 Laboratory for New Fiber Materials and Modern Textile, Growing Base for State Key Laboratory, Qingdao University, Qingdao 266071, China

**Keywords:** poly (methyl methacrylate), seed swelling polymerization, porous microspheres, high-performance liquid chromatography

## Abstract

Monodisperse cross-linked porous poly (methyl methacrylate) (PMMA) microspheres (~2.5 μm in diameter) were prepared by using an improved two-step seed swelling polymerization method with monodisperse micron-grade PMMA microspheres seeds. The porous PMMA microspheres with diverse surface morphology and pore structure were obtained by tuning porogen systems. The monodisperse porous PMMA microspheres, which were prepared using toluene:dibutylphthalate (DBP) = 1:1 (v/v) as a porogen system, had the smallest pore size and the largest specific surface area. Then, the monodisperse porous PMMA microspheres were subjected to high-performance liquid chromatography. The liquid chromatographic column filler successfully realized complete separation of arginine, glycine and glutamic acid, and the separation effect was good. The porous PMMA microspheres provide a new material for the separation of amino acids by liquid chromatography.

## 1. Introduction

Monodispersed porous polymer microspheres have a high specific surface area, good mechanical strength, uniform particle size and have been widely used in surface science-related fields such as adsorption separation, catalyst loading, ion exchange, sensors, solar cells and so on [1,2,3,4,5]. Methods for preparing porous polymer microspheres mainly include suspension polymerization [6,7], dispersion polymerization [8,9,10], emulsion polymerization [11,12], soap-free emulsion polymerization [13], precipitation polymerization [14,15], acid-base treatment [16,17,18,19] and seed swelling polymerization [20]. As the research progresses, the researchers find that these traditional methods have certain shortcomings. For instance, suspension polymerization can only synthesize microporous polymer microspheres, and the diameter and pore size of resulting microspheres are not uniform. Additionally, the microspheres prepared by the precipitation polymerization method have a low yield, and the solvent used in the preparation process has greater toxicity. The microspheres prepared by soap-free emulsion polymerization are prone to agglomeration. In order to get rid of the shortcomings of the traditional preparation methods, improved seed swelling and a dispersion polymerization method have been used. The seed swelling polymerization method utilizes the swelling process of polymer microspheres to construct the porous structure. The monodisperse polymer seed microspheres are prepared by the dispersion polymerization method. Afterwards, the monomers and inert solvent were added to promote swelling of the seed particles. Ultimately, monodisperse porous polymer microspheres were obtained. Swelling methods can be divided into the one-step swelling method, two-step swelling method, multi-step swelling method and dynamic swelling method [21,22,23].

Since the 1990s, scientists have prepared a variety of porous polymer microspheres by the seed swelling method. In 1993, Hosoya et al. [24] successfully prepared monodisperse porous polymer microspheres by the seed swelling method using two different monomers. They found that the performance of the microspheres prepared by the seed swelling polymerization method was better than that of the suspension polymerization method through chromatographic separation. Ma and coworkers [25] improved the seed polymerization method and successfully prepared monodisperse polymer porous microspheres with a water-soluble monomer. By adjusting the composition and ratio of monomers, porous microspheres with a high 2-hydroxyethyl methacrylate content and different morphologies were obtained. This research has successfully popularized the seed polymerization method and has made a great contribution to the progress of scientific research. In recent years, people have been committed to preparing cross-linked porous polymer microspheres with styrene [26,27,28]. Researchers successfully applied it to liquid chromatography column packing and found that it had very high column efficiency and good column stability [29,30,31,32].

Porogen, commonly inert and easy to remove from microspheres, plays a significant role in seed swelling polymerization. The type and amounts of porogen can affect the particle size and density of porous microspheres. EI-Aasser and coworkers [33] discussed the mechanism of two step seed swelling polymerization in porous microsphere preparation. By observing the structure of polymer particles and analyzing the kinetics of copolymerization, they proposed the pore forming mechanism of two step seed swelling polymerization in porous microsphere preparation. 

Poly (methyl methacrylate) (PMMA) microspheres have attracted wide attention in the field of high performance liquid chromatography. Okamoto et al. [34] prepared Poly (methyl methacrylate)s (PMMAs) with five diverse tacticities by radical or anionic polymerization. PMMA particles were used as high-performance liquid chromatography stationary phases. Aromatic hydrocarbons were separated via using polar eluents. Polar benzene derivatives were separated by nonpolar eluents. 

Intrinsic disorder proteins play an important role in the transcription regulation and signal transduction of proteins [35]. Glutamate and Arginine appear frequently in Intrinsic disorder proteins. Arginine is an important regulatory site for the function of disordered proteins. Glutamate is more common in alpha helices [36,37]. Therefore, it is of great significance to study arginine, glutamic acid and glycine. Lozanov et al. [38] established a high performance liquid chromatography method to separate amino acids after derivatization with *N*-(9-fluorenylmethoxycarbonyloxy)-succinimide (FMOC-Su). However, excessive reagents and their hydrolysates (FMOC-OH) still exist with major peaks in the chromatogram.

**Scheme 1:**
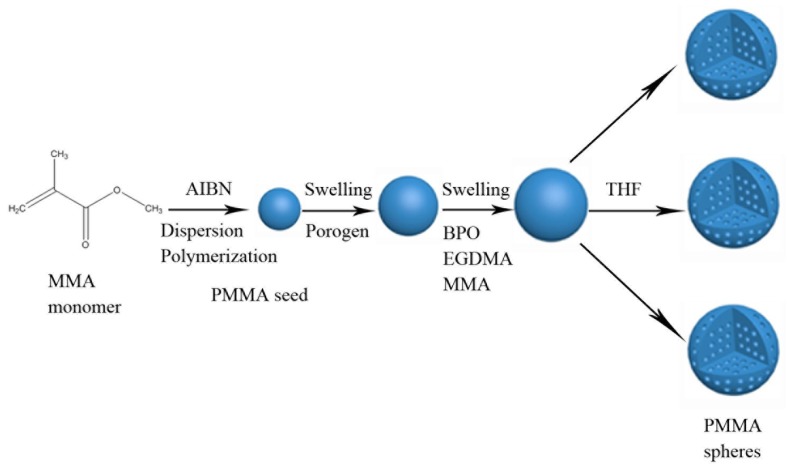
The mechanism of the two-step seed swelling method.

In our article, an improved two-step seed swelling polymerization method was used for preparing porous PMMA microspheres. This method was proposed by Ugelstad [39,40]. Here, different porogen systems were studied for adjusting microsphere properties, such as surface morphology and pore structure. Surface-activated seed microspheres were swelled with a water-insoluble swelling agent and porogen, and then a monomer and cross-linking agent were added for the second step of swelling. After the two-step swelling, polymerization was performed and monodisperse porous particles were obtained. The particle size of the microspheres prepared by this method was larger than that through the one-step swelling method. The microspheres we prepared could be used as a novel high-performance liquid chromatography filler. The mechanism of the two-step seed swelling method is shown in Scheme 1.

## 2. Materials and Methods 

### 2.1. Materials 

Methyl methacrylate (MMA, 99%), ethyleneglycol dimethacrylate (EGDMA, 99%) were purchased from Aladdin Chemical Regent Company, Shanghai, China. MMA and EGDMA were distilled under vacuum before use. Sodium dodecyl sulfate (SDS, 99%), azobisisobutyronitrile (AIBN, 98%), dibutylphthalate (DBP, 99%), toluene (99.5%), methanol (99.9%), ethanol (99.5%), acetonitrile (99.9%), hexane (98%), isopropanol (99.7%), tetrahydrofuran (THF, 99%) were purchased from Tianjin Chemical Company, Tianjin, China. Poly (*N*-vinylpyrrolidone) (PVP, Mn = 40,000) and polyvinyl alcohol (PVA-124, degree of polymerization 2400, degree of hydrolys is 98%) were provided by Sinopharm Chemical Reagent Company, Shanghai, China. Benzoylperoxide (BPO, 95%) was supplied from Tianjin Beichen Chemical Company, Tianjin, China. AIBN and BPO were recrystallized in ethanol before use. Arginine, Glycine and Glutamic acid were purchased from Aladdin Chemical Regent Company, Shanghai, China. Deionized water was purified by a Millipore framework (Milli-Q, Millipore, Suzhou, China).

### 2.2. Preparation of the polymethyl methacrylate seed particles 

Monodispersed micron-grade PMMA seeds were prepared by a typical dispersion polymerization process. The specific experimental procedures are as follows: Firstly, a certain amount of PVP K-30 was dissolved in a mixture of methanol and water (v/v = 7:3) in a three-necked flask, which accounts for 20% of the monomer fraction. Secondly, the reaction monomer methyl methacrylate was added to the three-necked flask.Thirdly, AIBN was added to the reaction system, which accounts for 2% of the monomer fraction. The whole reaction was carried out under a nitrogen atmosphere with a stirring speed of 300 r/min at 65 °C for 24 hours. Afterwards, the product was washed several times with deionized water and then ethanol. Finally, the resulting microspheres were dried in a vacuum oven.

### 2.3. Preparation of Monodispersed Porous PMMA Microspheres

Monodispersed porous PMMA microspheres with an average diameter of 2.5 μm were prepared by a modified two-step seed swelling polymerization. At the first swelling step, 0.26 g PMMA seeds were dispersed in 10 mL distilled water in a three-necked flask. After that, an emulsion that contained 20 mL distilled water, 0.075 g SDS, 1 mL DBP and 1 mL toluene was added to the three-necked flask. The emulsion was formed by sonication at 200 W for 1 h using an ultrasonic cell crushing apparatus (B1LON92-II, Shanghai Bilang Instrument Co., Ltd., Shanghai, China). Then, the solution was stirred at a mechanical agitation speed of 250 rpm at room temperature for 24 h. Another emulsion that contained 30 mL distilled water, 0.075 g SDS, 0.6 mL MMA, 0.6 mL EGDMA and 0.10 g BPO was added to the three-necked flask. The emulsion was also formed by sonication at 200 W for 1 h. The solution was stirred at a mechanical agitation speed of 250 rpm at room temperature for another 24 h. Afterwards, 3.5 mL of PVA aqueous solution with a concentration of 10 wt% was added to the three-necked flask. Polymerization of the monomer phase in the swollen seed particles was carried out at 70 °C for 24 h with a mechanical agitation speed of 300 rpm. After polymerization, the obtained particles were centrifuged and washed with ethanol three times. After that, the particles were extracted with THF at 60 °C for 12 h to completely remove the linear polymer and swelling agent. Eventually, the particles were centrifuged and washed with ethanol three times to form the monodispersed binary porous microspheres.

### 2.4. Characterization

The surface morphologies of the prepared PMMA seed microspheres and porous PMMA microspheres were characterized by scanning electron microscopy (SEM, JEOL JSM-6309LV, Japan Electronics Co., Ltd., Tokyo, Japan). Brunauer–Emmett–Teller tests were taken on an accelerated surface area and porosimetry analyzer (ASAP, Micromeritics 2020, Micromeritics Instrument Corporation, Shanghai, China). High-performance liquid chromatography (HPLC) experiments were performed using a HPLC (SEV P500) equipped with a UV detection system (SEV 500, Qingdao Rainbow Instrument Company, Qingdao, China).

### 2.5. Chromatography

The monodispersed porous PMMA microspheres were packed in a 75 × 4.6 mm I.D. stainless column using a chromatographic column packing machine at a pressure of 15 Mpa. Then, the column was attached to the HPLC system and rinsed with methyl alcohol at a flow velocity of 0.5 mL/min to balance the column until a constant UV base line appeared. The mobile phase was methyl alcohol:water = 1:9 (v/v) at a flow velocity of 0.5 mL/min at 25 °C. The UV detection wavelength was 254 nm.

## 3. Results and Discussion

### 3.1. Surface Morphology of Monodispersed PMMA Seed Microspheres

**Figure 1:**
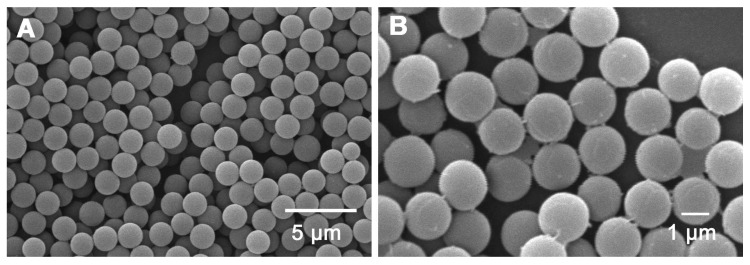
SEM images of PMMA seed microspheres prepared by dispersion polymerization.

The monodispersibility of the prepared PMMA seed microspheres was a guarantee for the monodispersibility of our final microspheres and the scanning electron microscopy (SEM) images are shown in Figure 1. From Figure 1, we can see that the seed microspheres are uniform and the particle size is about 1.7 μm. Narrow particle size distribution among the microspheres displayed good monodispersibility. The results indicated that monodispersed PMMA seed microspheres were successfully prepared, which could be further used to prepare monodispersed porous PMMA microspheres.

### 3.2. Effect of porogen systems on the preparation of monodispersed porous microspheres

**Figure 2:**
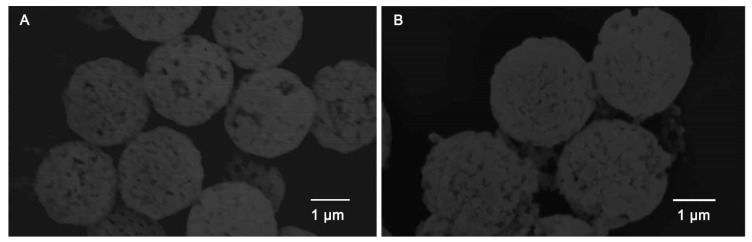
SEM images of PMMA porous microspheres prepared by the two-step seed swelling method. **A**: The porogen system was toluene:DBP = 1:1 (v/v), **B**: The porogen system was cyclohexanol:DBP = 1:1 (v/v).

As we mentioned above, the porogen system could significantly affect the surface morphology and properties of microspheres. Therefore, the effect of the porogen system on the fabrication of porous PMMA microspheres was investigated. After the two-step swelling treatment, the particle size of the PMMA microspheres is about twice as high as that of the seed microspheres (Figure 2). Figure 2A showed that the average diameter of cross-linked porous PMMA microspheres is about 2.5 μm. The surface of the microspheres is rough, and the pores are uniformly distributed on the surface of the microspheres. From Figure 2B, we found that the microspheres have a larger particle size of about 2.8 μm, and the pores are densely distributed on the surface of the microspheres. However, there are small impurities around the microspheres and the microspheres were partially collapsed, indicating that the microspheres had lower mechanical strength. 

**Figure 3:**
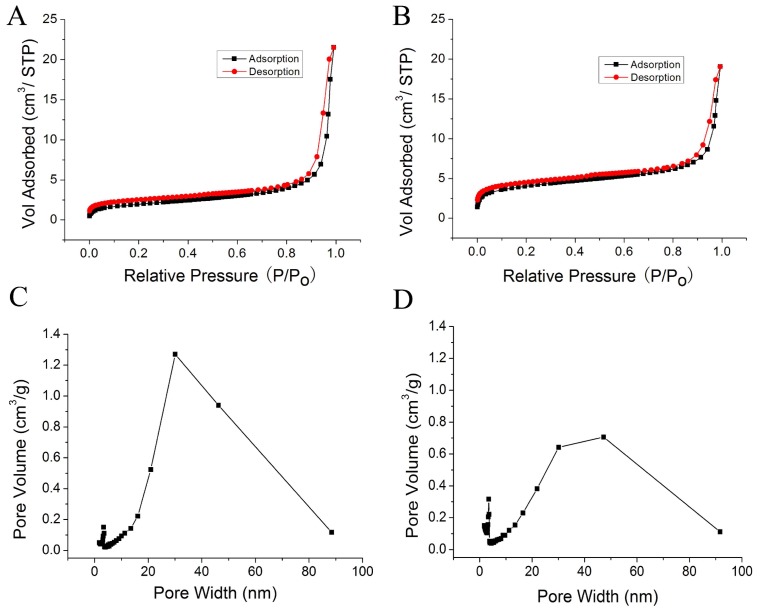
Nitrogen adsorption and desorption isothermal curves and pore size distribution curves of monodispersed PMMA-EGDMA porous microspheres. **A**, **C**: The porogen system was toluene:DBP = 1:1 (v/v); **B**, **D**: The porogen system was cyclohexanol:DBP = 1:1 (v/v).

The surface pore distribution and nitrogen adsorption and desorption isotherm curves of the two porous microspheres were detected by Brunauer-Emmett-Teller (BET). Figure 3A and Figure 3B demonstrated that the isothermal curves of the microspheres are consistent with the fifth type of reversible adsorption isothermal curves, indicating that the adsorption and desorption processes of these two microspheres were carried out through the surface porous structure of the microspheres. Figure 3C showed that the porous microspheres prepared by using toluene:DBP = 1:1 (v/v) as the porogen system have a large specific surface area and uniform surface pore distribution with an average pore diameter of 24.4 nm, whereas the porogen system was replaced with cyclohexanol:DBP = 1:1 (v/v), and the porous microspheres displayed a larger pore size of 16.5 nm and lower mechanical strength (Figure 3D). Thus, in this study, the toluene:DBP = 1:1 (v/v) was an ideal porogen system for microspheres. The effect of the ratio of toluene and DBP on the preparation was further studied.

**Figure 4:**
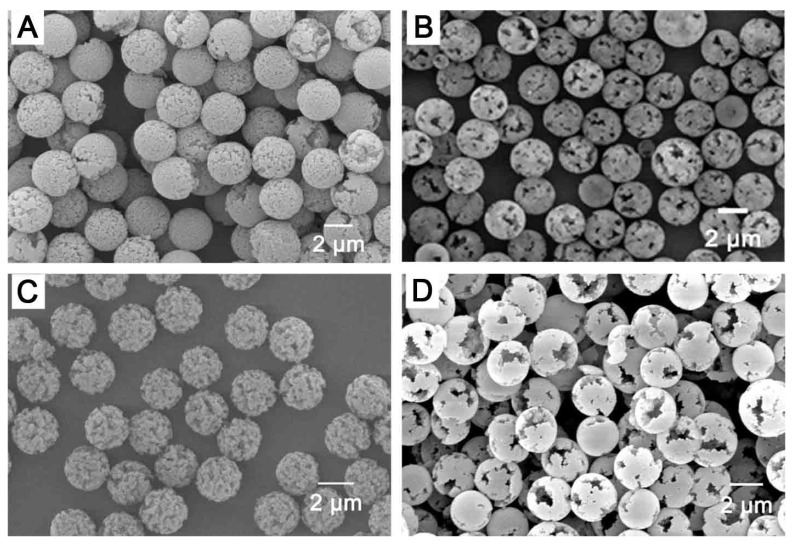
SEM images of porous PMMA microspheres prepared by two-step seed swelling method. **A**: The porogen system was Toluene:DBP = 1:1 (v/v); **B**: The porogen system was Toluene:DBP = 1:2 (v/v); **C**: The porogen system was Toluene:DBP = 1:0 (v/v); **D**: The porogen system was Toluene:DBP = 0:1 (v/v).

The effect of different proportions of toluene and DBP on the preparation of PMMA porous microspheres was emphatically discussed. From Figure 4A to D, the composition of porogen was toluene:DBP = 1:1 (v/v), toluene:DBP = 1:2 (v/v), toluene:DBP = 1:0 (v/v) and toluene:DBP = 0:1(v/v), respectively. It can be seen that the average size of PMMA microspheres after the two-step swelling was about 2.5–3.1 μm. Nevertheless, the surface morphology of the microspheres was different among these four systems. Figure 4A showed that the porous PMMA microspheres had an average pore size and uniform pore distribution, which was smaller than the ones formed by other porogen systems. When the proportion of DBP in the porogen was increased, because of the greater volatility and the water extraction of DBP, phase separation of the microspheres occured during swelling and polymerization, resulting the uneven distribution of DBP within the microspheres (Figure 4B). In Figure 4C, only toluene was used as a porogen. Toluene is poorly water-soluble and has large droplets during the swelling process. Therefore, the resulting porous microspheres have larger pores with the pore size reaching hundreds of nanometers. In Figure 4D, only DBP was used as the porogen. The inside of the microspheres was completely washed out due to the water extraction of DBP, resulting the internal hollow shell structure. From the above analysis, it can be concluded that the porogen system with toluene: DBP of 1:1 (v/v) was the ideal porogen system. Monodispersed porous PMMA microspheres with larger specific surface area can be obtained. Therefore, the microspheres prepared using this porogen system were further tested as a filler for a high-performance liquid chromatography column. 

### 3.3. Application of Liquid Chromatography of Monodispersed PMMA Porous Microspheres

**Figure 5:**
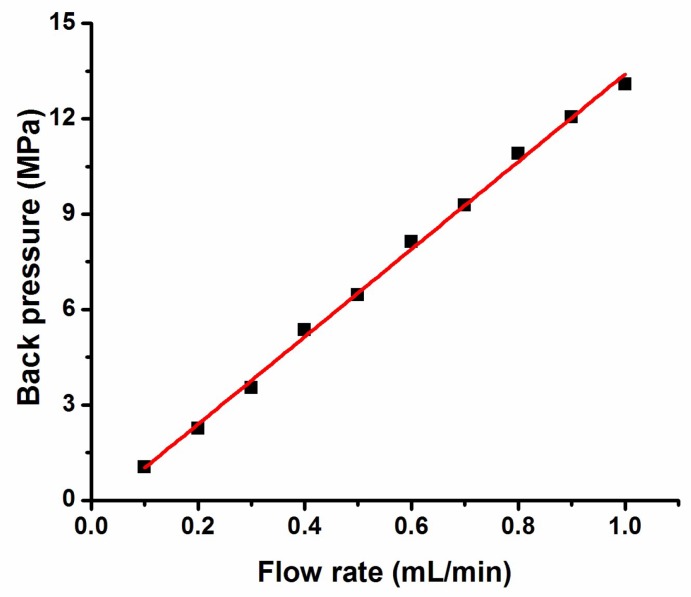
The relationship between the flow rate and the column pressure of the monodispersed PMMA porous microspheres stationary phase prepared by using toluene:DBP = 1:1 (v/v) as porogen system. Column: 75 × 4.6 mm; mobile phase: methanol:water = 1:9 (v/v); temperature: 25 °C.

The monodispersed PMMA microspheres with uniform particle size were packed in a 75 × 4.6 mm I.D. column using the chromatographic column packing machine. The liquid chromatography separation performance of the column packings for three kinds of amino acids was studied. The mobile phase is methanol:water = 1:9 (v/v). First, the column pressure was measured at different flow rates and the results are shown in Figure 5. As can be seen from Figure 5, the column pressure was gradually increased with the increase of the flow rate. The column pressure had a linear relationship with the flow rates (P = −0.3586 + 13.7612x (R^2^ = 0.9977)). It indicated that the column was stable with the increasing flow rate of the mobile phase.

**Figure 6:**
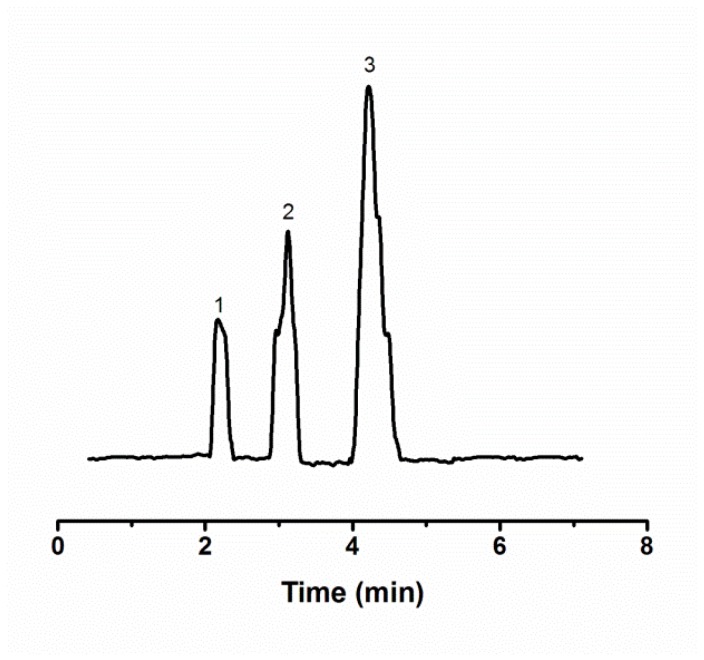
Separation of amino acids on the PMMA column. Column dimensions: 75 × 4.6 mm I.D.; mobile phase: methanol:water = 1:9 (v/v); temperature: 25 °C; flow rate: 0.5 mL/min; elution order: 1 Arginine, 2 Glycine, 3 Glutamic acid.

The chromatographic separation performance of the porous PMMA microsphere column packing was investigated by separating a mixture of amino acids at room temperature under the flow rate of 0.5 mL/min using a methanol-water mixture (v/v = 1:9) as an eluent. Using the hydrophobic effect of PMMA materials, amino acids can be successfully separated on HPLC because of the different hydrophobicity degrees of amino acids. Figure 6. showed that arginine was first isolated after 2 min of injection, but its peak was weaker. Then, glycine was separated at 3 min; 1 min later, glutamic acid was also completely separated and its peak was stronger. It indicated that the column can successfully separate these three amino acids and achieve complete separation. In addition, the column length was only 75 mm, which was shorter in size. It showed an excellent separation effect with less filler. It demonstrated that the porous PMMA microspheres have excellent separation properties.

Table 1 shows the retention time relative standard deviation (RSD) of the three amino acids in the PMMA (toluene:DBP = 1:1 (v/v)) stationary phase. After 5 consecutive separations, the retention time RSD of the three amino acids was less than 1%. The retention time RSD of the three amino acids was less than 2.0% after 7 days repeated operation on the same column. After the separation of the same packing in the five different columns, the retention time RSD of the three amino acids was less than 3.0%. After about 200 consecutive injections, the retention time RSD of the three amino acids was around 2.0%. These results indicated that the prepared porous PMMA microspheres have good reproducibility and stability when they are used as liquid chromatographic column packing. After a long period of separation, the separation performance did not decrease obviously. Thus, it could be used as stable chromatographic column packing and can be widely used in analytical separations.

## 4. Conclusions

Monodispersed cross-linked porous PMMA microspheres (~2.5 μm in diameter) were successfully prepared by the improved two-step seed swelling seed polymerization method. The effects of different porogens on the seed swelling process were studied. The porous PMMA microspheres with diverse surface morphology and pore structure were obtained by regulating the porogen systems. While using toluene:DBP = 1:1 (v/v) as the porogen system, the microspheres had better monodispersity and larger specific surface area. Afterwards, the monodispersed porous PMMA microspheres were subjected to high-performance liquid chromatography. The liquid chromatographic column packing successfully achieved the complete separation of arginine, glycine and glutamic acid. It is different from the conventional column packing, which effectively broadens the chromatographic detection range. Hence, it provides new materials for the separation of amino acids by high-performance liquid chromatography.

## Figures and Tables

**Table 1:** The retention time relative standard deviation of porous PMMA microsphere filler and retention times of three amino acids.

Retention time RSD (%)				
Amino acid	Run to run (n = 5)	Day to day (n = 7)	Column to Column (n = 5)	Continuous 200 times running
Argnine	0.96	1.82	2.56	2.05
Glycine	0.89	1.78	2.31	1.93
Glutamic acid	0.93	1.90	2.48	2.01
